# CIS1/336: Use of a Database Program to Convert Laboratory Information System (LIS) Data to Intranet Web Pages

**DOI:** 10.2196/jmir.1.suppl1.e6

**Published:** 1999-09-19

**Authors:** L Massey

**Affiliations:** ^1^University of Saskatchewan, SaskatoonCanada

**Keywords:** Clinical Laboratory Information Systems, Hospital Information Systems, Database Systems

## Abstract

**Introduction:**

The composition of Laboratory Ward Manuals is a time-consuming exercise. To keep manually prepared books up-to-date requires constant effort, amounting to several days or weeks of activity every year. As most of this information is found in the various tables of the usual Laboratory Information System (LIS), it should be possible to reformat this data into a form suitable for publishing on paper or on Web Pages. When the three hospitals in Saskatoon became part of a single operating entity, it became a necessity to redo the procedure manuals and ward manuals for all of the laboratories. As part of this exercise, we sought ways to minimize the work required. One method was to automate the ward manuals. Accordingly, an automated way of producing an Intranet ward manual was devised to reduce the work of the laboratory staff in this continuing endeavour.

**Methods:**

The query language of the SoftLab LIS (SCC Clinical Information Systems, Palm Harbor, Fla, USA) was used to extract standard test data, including various descriptive terms, specimens required, minimum volumes, reference ranges, &c, &c in a comma-delimited form for input to a database application program. Paradox 8 (Corel Corporation) was used to build the several files necessary to hold the various forms of data from the many databases of the LIS. Paradox Macros were written to combine records from the various files into a single record containing concatenated strings to format the appropriate data in a form, which would display in a single column in the ultimate report. Using the standard Paradox report forms, HTML Web pages could then be generated which became available for publishing on the Hospital Intranet.

**Results:**

This procedure was used with the Saskatoon LIS to produce Intranet Web Pages, and also a print version of the Ward Manual). Once the Paradox Macros have been debugged, preparation of the updated Web Pages takes only a few minutes, and updates can be incorporated almost completely automatically.

**Discussion:**

Spread Sheet (SS) and Database (dB) Handlers have become highly flexible tools for generating many different types of reports, from almost any form of data. With the addition of HTML generation capabilities in the standard dB and SS application programs this becomes an even easier matter. This puts highly sophisticated Web Page generation capabilities into everyone's hands.

